# Primary Cutaneous Gamma–Delta T-Cell Lymphoma Presenting With Hemophagocytic Lymphohistiocytosis in a Young Polynesian Male

**DOI:** 10.1155/crh/8582804

**Published:** 2025-05-23

**Authors:** Saeed Arabi, Victoria Vardell, Timothy Hanley, Scott Florell, Ahmad Halwani, Ming Lim

**Affiliations:** ^1^Department of Internal Medicine, University of Utah, Salt Lake City, Utah, USA; ^2^Division of Hematology and Hematologic Malignancies, Huntsman Cancer Institute, University of Utah, Salt Lake City, Utah, USA; ^3^Division of Hematopathology, Department of Pathology, University of Utah, Salt Lake City, Utah, USA; ^4^Department of Dermatology, University of Utah, Salt Lake City, Utah, USA

**Keywords:** adipocyte rimming, brentuximab vedotin, hemophagocytic lymphohistiocytosis, panniculitis, primary cutaneous T-cell lymphoma

## Abstract

Primary cutaneous gamma–delta T-cell lymphoma (PCGD-TCL) is a very rare subtype of cutaneous T-cell lymphoma. We report the case of a young Polynesian male who presented with fever and an abdominal wall rash and highlight the workup leading to the diagnosis of PCGD-TCL. As PCGD-TCL is rare and mimics other medical conditions, its diagnosis requires a high index of suspicion and can be challenging. Hemophagocytic lymphohistiocytosis (HLH) occurs with PCGD-TCL and can be a marker of more invasive disease. There are no well-defined treatment guidelines, but the most common treatment approach is anthracycline-based multiagent chemotherapy followed by allogeneic stem cell transplant. Targeted therapies are being increasingly used as well. Prognosis remains poor and 5‐year survival is < 20%, particularly in more invasive disease. We highlight how this patient's demographic varies from the published literature and discuss some unique particulars of the diagnostic evaluation and treatment, especially in the presence of concurrent HLH.

## 1. Introduction

Primary cutaneous gamma–delta T-cell lymphoma (PCGD-TCL) is an exceedingly rare and aggressive disease that represents < 1% of the cutaneous T-cell lymphomas. Due to the scarcity of published evidence, there is no well-defined guidance on diagnosis or treatment, and existing data consist mainly of case reports and series. We report the case of a young Polynesian male who was diagnosed with PCGD-TCL after presenting with fever and an abdominal wall rash. This case report describes PCGD-TCL in a patient younger than typically reported in the literature [1], offering insight into its presentation, diagnostic challenges, and management in a younger demographic. Given the rarity and aggressive nature of PCGD-TCL, this report enhances clinical awareness, aids in early recognition, and informs treatment decisions.

## 2. Case

A 28-year-old previously healthy male with no past medical history presented to a local hospital with fever that had been ongoing for over a week. His fevers reached up to 40.6°C (105°F) and were occurring daily with no cyclical pattern. He also noted an area of hardness on the right side of his abdomen with no accompanying pain or overlying skin changes. After a week of persistent high-grade fever, he developed emesis with fluid intake, dizziness, and lightheadedness, which led him to seek medical evaluation. Other than acetaminophen and ibuprofen as needed, he was not taking any prescribed or over-the-counter medications. He had no family history of hematologic malignancy or other medical conditions of concern. He had no history of tobacco or illicit substance use, had rare alcohol consumption, and was in a monogamous sexual relationship with a female partner. He was a laborer at a local city facility. He was born in the Pacific Islands but had been living in the continental United States with no travel in the past year.

At presentation, his temperature (T) was 39.2°C, blood pressure (BP) was 103/59, heart rate (HR) was 116, respiratory rate (RR) was 23, and peripheral capillary oxygen saturation (SpO_2_) was 90% on room air (RA). Cardiopulmonary examination was unremarkable. Abdominal examination was remarkable for right lower quadrant erythema associated with mild tenderness and warmth. There was no guarding, rebound tenderness, fluctuance, or crepitus. His initial laboratory workup (Table 1) revealed a normal white blood cell (WBC) count, a microcytic anemia, hyponatremia, hypokalemia, as well as elevated serum creatinine, serum aminotransferases, lactic acid, and creatine kinase (CK). A computed tomography scan of the abdomen and pelvis revealed cellulitis of the right lateral abdominal wall and flank, with no soft tissue gas or fluid collection, and also revealed hepatomegaly.

The patient was admitted and started on empiric antibiotic therapy for treatment of cellulitis. His fever and erythema persisted over the next week despite multiple broad-spectrum antibiotic regimens, and he remained hemodynamically stable. Blood and stool cultures were negative. A urinalysis, stool *Clostridioides difficile* polymerase chain reaction (PCR), respiratory viral panel, human immunodeficiency virus (HIV) screen, serologies for Epstein–Barr virus (EBV), cytomegalovirus (CMV), and herpes simplex virus (HSV) were unremarkable. Antinuclear antibody (ANA), rheumatoid factor (RF), and cytoplasmic and perinuclear antineutrophilic cytoplasmic antibodies (c-ANCA and p-ANCA) were all negative. An echocardiogram showed no valvular vegetations. These findings, together with the discordantly low normal WBC count, raised concern for an invasive fungal, mycobacterial, autoimmune, or possibly a hematologic process. A dermatology consult was recommended for a skin biopsy, and arrangements were made to transfer the patient to our hospital for an inpatient rheumatology evaluation.

**Figure 1:**
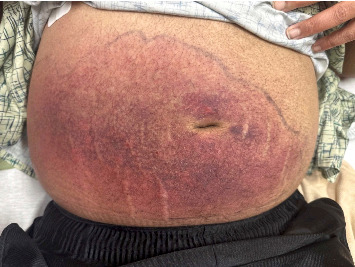
Large violaceous contiguous patch spreading over the anterior and right side of the abdominal wall.

The patient was transferred to our hospital 11 days after his initial presentation. Upon presentation, his vital signs were T 39.2, BP 128/88, HR 68, RR 18, and SpO_2_ 97% on RA. His physical examination revealed the same right lower abdominal wall erythema and was, otherwise, similar to his original presentation (Figure 1). Laboratory studies at transfer (Table 1) revealed resolution of hyponatremia, hypokalemia, and acute renal insufficiency but worsening of microcytic anemia and persistence of lactic acidosis as well as elevated serum aminotransferases and CK. Further studies revealed a serum ferritin of 40,670 ng/mL and a lactate dehydrogenase (LDH) of 2197 U/L. These findings, considered in the context of his history, raised concern for hemophagocytic lymphohistiocytosis (HLH) or macrophage-activating syndrome (MAS). Studies were sent for triglycerides, fibrinogen, soluble interleukin 2 receptor (sIL-2r), and C-X-C Motif Chemokine Ligand 9 (CXCL9), all of which were abnormal (Table 1). The patient's HScore was 235, conferring 98%-99% probability of HLH. He also met > 5 of the HLH-2004 criteria. Treatment was started with dexamethasone 10 mg/m^2^ of body surface area.

**Figure 2:**
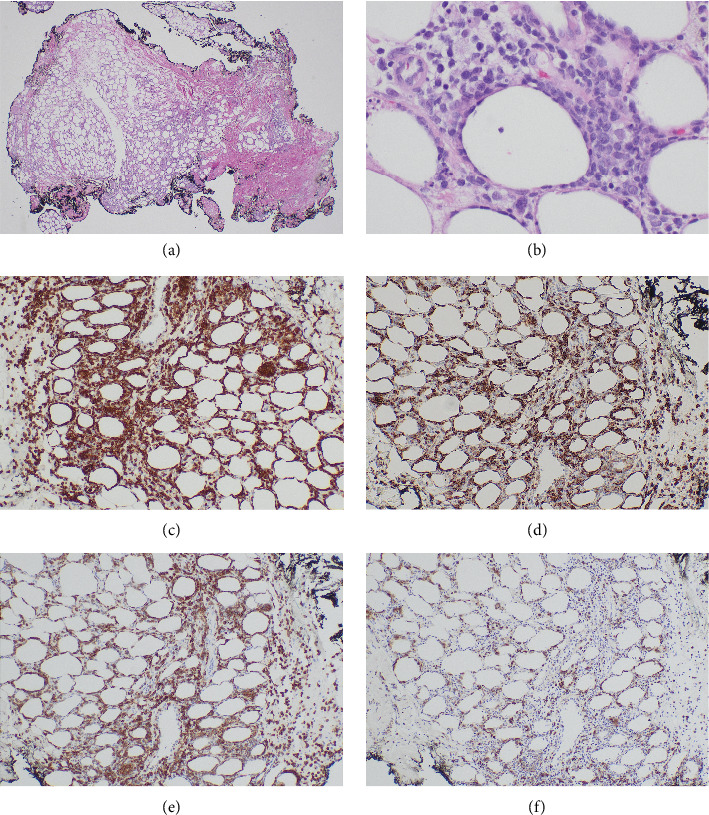
Skin biopsy findings. Low magnification ((a), 20x) image showing a lobular, pannicular infiltrate of large lymphoid cells with pleomorphic nuclei and irregular nuclear contours that focally rim adipocytes ((b), 400x). Focal hemophagocytic lymphohistiocytosis is also noted. Tumor cells show immunostaining for CD3 ((c), 100x), CD8, with rimming of adipocytes ((d), 100x), and TCR-delta ((e), 100x). The BF-1 shows staining of small lymphocytes ((f), 100x).

**Figure 3:**
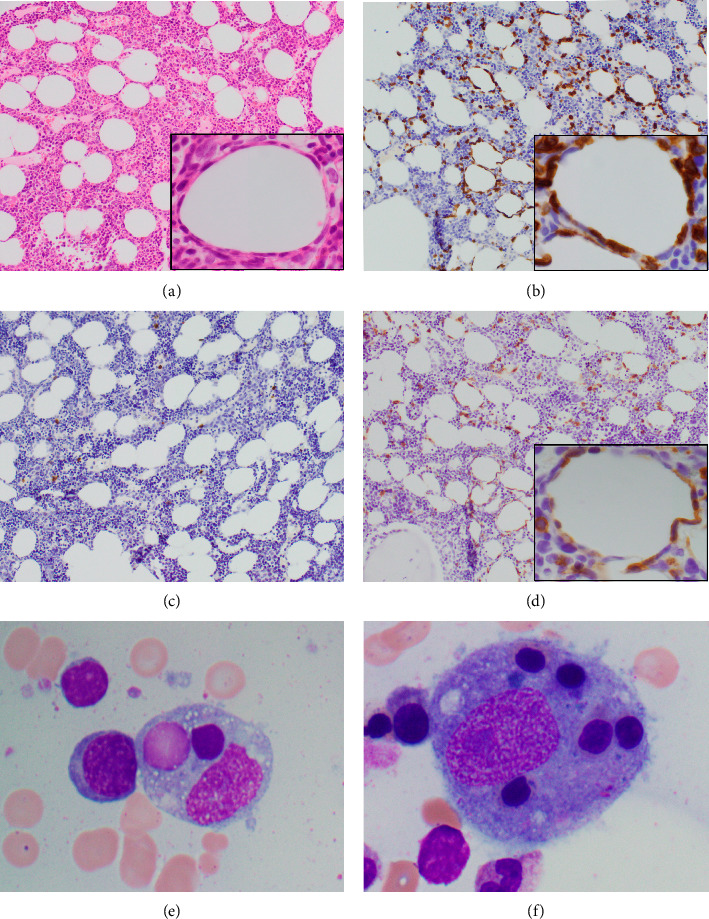
Bone marrow biopsy findings. Bone marrow trephine biopsy at 20X magnification stained with hematoxylin and eosin (a), CD3 (b), CD20 (c), and γδ TCR (d), demonstrating rimming of fat by γδ T cells. Insets in (a), (b), and (d) at 100X magnification. Bone marrow aspirate at 100X magnification demonstrating extensive hemophagocytosis (e-f).

**Figure 4:**
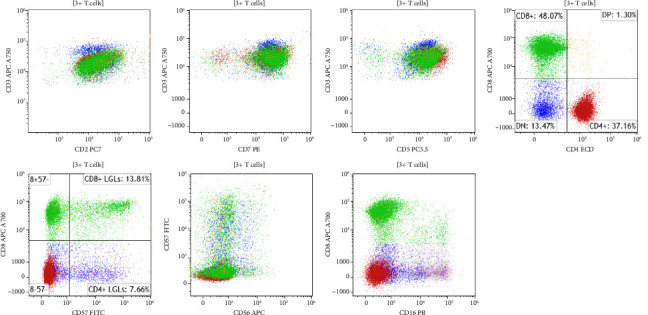
Flow cytometric analysis of CD3+ T cells from the bone marrow aspirate specimen. Green, CD8+ T cells. Red, CD4+ T cells. Blue, CD4-CD8- γδ T cells. The γδ T cells express bright CD3, normal levels of CD2, CD5, and CD7, partial CD56, partial CD57, and partial CD16. No definitive phenotypic abnormalities were detected by flow cytometry.

A skin biopsy was obtained from the patient's abdominal wall erythema shortly after arrival at our hospital and was preliminarily read as panniculitis, but T-cell lymphoma could not be ruled out. Thus, a second skin biopsy was obtained 3 days later. A bone marrow biopsy was also obtained. The biopsies from skin and bone marrow both confirmed the diagnosis of a panniculitic pattern gamma–delta primary cutaneous T-cell lymphoma with bone marrow involvement (Figures 2, 3, and 4). Immunohistochemistry staining of the skin biopsy for CD30 revealed weak staining of about 1%-2% of lymphocytes.

The patient was transferred to the inpatient hematology service and started on a CHOED chemotherapy regimen consisting of cyclophosphamide, doxorubicin hydrochloride, vincristine, etoposide, and dexamethasone (instead of prednisone in the setting of HLH). He completed the first cycle of treatment while hospitalized. He was then discharged on a dexamethasone taper and completed six cycles of this regimen on an outpatient basis. A subsequent bone marrow biopsy showed complete remission (CR). He was then started on BV as a bridge to allogeneic stem cell transplant (allo-SCT). At 6 months following the diagnosis, he remains alive and is undergoing evaluation for allo-SCT.

## 3. Discussion

PCGD-TCL is a rare subtype of primary cutaneous lymphoma (PCL), accounting for less than 1% of PCLs. We report the case of a 28-year-old male diagnosed with PCGD-TCL after presenting with abdominal rash and HLH. Although there have been multiple reports of PCGD-TCL in younger patients [2–7], the majority of reported cases tend to be older, with a median age of diagnosis between 50 and 60 years [1]. Males and females are equally affected in general [1]. Data on race and ethnicity are not commonly reported.

### 3.1. Clinical Features and Diagnosis

PCGD-TCL typically presents on the extremities as plaques, nodules, or tumors, which could be associated with ulceration and/or necrosis. It can also affect sites other than the extremities [8]. Importantly, PCGD-TCL can also present as erythema and mimic nonspecific panniculitis [9]. As the presenting features of PCGD-TCL can mimic that of many other malignant and nonmalignant disorders, its diagnosis is frequently challenging and requires correlation between clinical, laboratory, and histologic features. A high index of suspicion needs to be maintained to make the correct diagnosis.

Biopsy of the skin is of particular importance in this context and should be performed early on whenever there is ambiguity about the diagnosis or when a diagnosis is apparent but the response to treatment is not as expected. As with other cutaneous T-cell lymphomas, it is common to need multiple skin biopsies to make a diagnosis of PCGD-TCL, as in this case [10, 11].

The identification of HLH in this patient greatly accelerated the diagnostic workup toward the eventual identification of PCGD-TCL. HLH has been reported in up to 45% of the patients with PCGD-TCL [7, 12, 13] and may signify more aggressive disease. HLH is defined by the HLH-2004 criteria [14], and the HScore can also be calculated to predict the probability of HLH [15]. In this patient, persistent fevers, hepatomegaly, and bicytopenias were present upon transfer, and a ferritin that was originally ordered as part of microcytic anemia workup was markedly elevated, raising the suspicion further.

HLH can be primary or secondary, with primary HLH affecting pediatric populations predominantly [16]. Although this patient is rather young, secondary HLH is much more likely in an adult patient. A finding of secondary HLH necessitates a workup for the underlying etiology, which is most commonly a viral infection (especially EBV), a rheumatologic condition or a hematologic malignancy [17].

### 3.2. Pathology

The fifth edition of the WHO Classification of Haematolymphoid Tumors published in 2022 recognized PCGD-TCL as a separate entity after it was included provisionally under “primary cutaneous peripheral T-cell lymphoma, rare subtypes” in prior versions of the classification [18, 19]. PCGD-TCL can involve the epidermis, dermis, subcutaneous tissues, or any combination thereof. The particular skin layers involved are associated with differences in clinical course and in prognosis among patients with PCGD-TCL, as infiltration of deeper skin layers typically signifies more aggressive disease and worse prognosis [20].

Subcutaneous infiltration with rimming of fat cells was seen in this patient (Figure 2). Fat cell rimming is a feature common to both subcutaneous PCGD-TCL and subcutaneous panniculitis-like T-cell lymphoma (SCPTL). The key difference is that the latter is a lymphoma derived from α/β T cells and is associated with a more favorable prognosis. The two can be distinguished using immunohistochemistry staining for γ/δ TCR.

Although PCGD-TCL frequently spreads to the mucosa and extranodal sites, it does not commonly involve lymph nodes, the spleen, or the bone marrow. Bone marrow involvement is rarely reported in published case reports, and different case series report incidence of bone marrow involvement ranging from 0% to 20% [4, 12, 21]. Bone marrow involvement also tends to accompany HLH (Figure 3) [4, 20]. Bone marrow involvement can be considered a marker of aggressive disease; however, there are no clear data on its prognostic implication.

### 3.3. Prognosis

Multiple cases reports and series in the literature concur that PCGD-TCL is associated with a poor prognosis. The 5-year survival rate is generally under 20% [2, 3, 22] and median overall survival ranges from 15 to 31 months [9]. A recent National Cancer Database (NCDB) study demonstrated a 1-year survival rate of 61% and a 3-year survival rate of 47% [9].

As mentioned earlier, histopathologic features influence prognosis. Specifically, epidermotropic lymphomas are generally associated with better survival than their dermal or subcutaneous (panniculitis-like) counterparts [1–3, 22]. The subcutaneous involvement in this patient is, therefore, a poor prognostic finding.

Although there are no data specifically about patients of Pacific Islander descent, there are data suggesting that race and socioeconomic factors appear to influence survival rates in PCGD-TCL [9]. For instance, Black Americans tend to have a lower overall survival rate at 3 years (37.5%) compared with White Americans (48.9%). In addition, patients with lower incomes (< $48,000/year) have a lower 3-year survival rate (27.6%), compared with high-income patients (49.5%). Government insurance is also associated with decreased survival at 3 years (38.8%) compared with private insurance (58.1%). Of note, in the study by Ashok Kumar et al., some of these comparisons did not achieve statistical significance, possibly due to the small sample size.

More recently, a case series by Foss et al. showed an encouraging 3-year overall survival of 70% [21]. In the NCDB-based study by Ashok Kumar et al., PCGD-TCL patients with early disease who did not require chemotherapy or radiation had a 3-year survival rate of 74.3%. These patients may have received topical therapy (e.g., with corticosteroids), although this information is not captured in NCDB, making it difficult to interpret this figure [9].

### 3.4. Treatment

Due to its rarity, PCGD-TCL is often included in trials that include several lymphoma subtypes. This creates challenges interpreting evidence regarding treatment efficacy in PCGD-TCL patients.

The most commonly reported frontline therapies are anthracycline-based regimens such as cyclophosphamide, doxorubicin hydrochloride, vincristine, and prednisone (CHOP) and etoposide + CHOP (EPOCH) [1]. However, the outcomes are generally suboptimal as only 20%–30% of the patients achieve CR with treatment [1, 3, 5, 21]. Given his concurrent HLH, our patient was treated with a modified CHOED regimen, which substitutes dexamethasone for prednisone in a CHOP regimen and adds etoposide [16].

As with other peripheral T-cell lymphomas, targeted therapies have recently sparked interest as potential treatments in PCGD-TCL. Few studies investigated these treatments specifically in PCGD-TCL, and much of the evidence comes from other types of peripheral T-cell lymphomas. An example of targeted therapies used in PCGD-TCL is brentuximab vedotin (BV), an anti-CD30 monoclonal antibody that has been described in multiple case series and reports [23–28]. Interestingly, no correlation has been demonstrated between efficacy of BV and the level of CD30 expression [29–31]; therefore, even patients with low CD30 expression like our patient have benefited from treatment with BV. Other examples of targeted therapies include mogamulizumab (anti-CXCR4) [32] and alemtuzumab (anti-CD52) [33, 34]. These targeted therapies and others remain an ongoing area of investigation [1].

Stem cell transplantation (most commonly allogeneic) has been used in patients with PCGD-TCL in first remission following multiagent chemotherapy. Though no large clinical trials exist to study its efficacy, some cases series have reported encouraging outcomes in these patients [7, 23].

## 4. Conclusion

This case report describes some of the challenges related to the diagnosis and treatment of PCGD-TCL. It highlights that rare malignancies like PCGD-TCL should be considered in patients with unexplained fever and especially in patients with newly identified HLH. Multiagent chemotherapy is the first-line treatment. The 5-year survival is generally low. However, newer targeted therapies and stem cell transplantation may prolong disease remission and improve survival in these patients.

## Figures and Tables

**Table 1:** Laboratory values at initial presentation and upon transfer to our hospital.

Laboratory test	Value at presentation	Value at transfer (D + 11)	Reference range
White blood cell (WBC) count	4.4 × 10^3^/μL	4.36 × 10^3^/μL	4.3–11.3 × 10^3^/μL
Hemoglobin	12.5 g/dL	9.0 g/dL	14.8–17.8 g/dL
Hematocrit	37.5%	28.5%	44.2%–53.0%
Platelets	281 × 10^3^/μL	165 × 10^3^/μL	159–439 × 10^3^/μL
Absolute neutrophil count (ANC)	2.91 × 10^3^/μL	2.6 × 10^3^/μL	2.0–7.4 × 10^3^/μL
Mean corpuscular volume (MCV)	74.4 fL	75.4 fL	81.2–96.6 fL
Sodium	129 mmol/L	136 mmol/L	136–144 mmol/L
Potassium	3.2 mmol/L	3.8 mmol/L	3.3–5.0 mmol/L
Chloride	98.8 mmol/L	106 mmol/L	102–110 mmol/L
Carbon dioxide	21 mmol/L	18 mmol/L	20–26 mmol/L
Blood urea nitrogen (BUN)	14 mg/dL	17 mg/dL	8–24 mg/dL
Creatinine	1.3 mg/dL	0.71 mg/dL	0.72–1.25 mg/dL
Glucose	127 mg/dL	113 mg/dL	64–128 mg/dL
Aspartate aminotransferase (AST)	460 U/L	225 U/L	16–40 U/L
Alanine aminotransferase (ALT)	261 U/L	114 U/L	5–60 U/L
Total bilirubin	0.6 mg/dL	0.5 mg/dL	0.2–1.4 mg/dL
Alkaline phosphatase	120 U/L	83 U/L	38–126 U/L
Albumin	3.2 g/dL	2.7 g/dL	3.5–5.0 g/dL
Lactic acid	2.5 mmol/L	2.6 mmol/L	0.5–2.2 mmol/L
Total creatine kinase (CK)	2547 U/L	1874 U/L	20–200 U/L
Ferritin		40,670 ng/mL	30–400 ng/mL
Lactate dehydrogenase (LDH)		2197 U/L	100–253 U/L
Triglycerides		985 mg/dL	30–149 mg/dL
Fibrinogen		77 mg/dL	150–430 mg/dL
Soluble interleukin 2 receptor (sIL-2R)		7266 pg/mL	175.3–858.2 pg/mL
C-X-C motif chemokine ligand 9 (CXCL9)		573,832 pg/mL	≤ 647 pg/mL

## Data Availability

Data sharing is not applicable to this article as no datasets were generated or analyzed during the current study.
